# Stock keeping accuracy: A data based investigation of storage tank calibration challenges

**DOI:** 10.1016/j.dib.2018.06.122

**Published:** 2018-07-05

**Authors:** Aderibigbe Israel Adekitan, Osemwegie Omoruyi

**Affiliations:** Electrical and Information Engineering, Covenant University, Ota, Nigeria

**Keywords:** Oil and fuel, Transportation, Storage tank, Calibration accuracy, Data pattern recognition, Stock Accounting

## Abstract

In fuel dispensing and fuel haulage companies, adequate stock tracking is mandatory for performance and business productivity analysis. Stock monitoring is vital for inventory management; it is a tool that enables adequate planning in terms of importation requirements when stock is low and for general price management. The accuracy of stock inventory depends largely on the accuracy of the calibration data of the various storage tanks and structures deployed along the value chain. Mobile tanks are prone to harsh conditions due to poor road networks in some countries which affect tanker truck alignment and suspension systems, and all these affects tank calibration accuracy. This is further aggravated by various road impacts, and accidents that sometimes distort portions of the tank shape making it to lose its cylindrical profile in some sections. Excessive stock variations is often linked to product theft and sabotage, though this may be true in some instances, but at times, this variations may be as a result of inaccuracies in tank calibration. The dataset presented in this paper contains tank calibration parameters for two consecutive calibrations carried out on the same mobile storage tank. The statistical analysis attempts to identify variations between the two tank calibration dataset as an indication of potential stock accuracy variations.

## Specifications Table

**Table:** 

Subject area	*Engineering, Stock Accounting*
More specific subject area	*Petrochemical Engineering, Stock keeping, pattern recognition*
Type of data	*Table, graph, figures and spread sheet file*
How data was acquired	*Dataset acquisition from the calibration chart log for a mobile fuel storage tank*
Data format	*Raw, analyzed*
Experimental factors	*Data was extracted on three (3) key tank calibration parameters; the tank dip, the cumulative volume and the volume increment. The data is based on charts for 2 successive tank calibrations performed within a 3-year period*
Experimental features	*Frequency distributions, Linear regression models and Generalized linear model analysis were carried out to identify pattern variations between the two calibration data sets for the same tank*
Data source location	*Fuel haulage company in Nigeria*
Data accessibility	*The dataset is available in a spreadsheet file attached to this article*

## Value of the data

• The data set contains fuel storage tank calibration parameters. These are important parameters that are stored on software platforms for automatic computation of fuel stock, and this enables stock reconciliation, product loss tracking and profit accounting.
• The availability of this data, and the analysis presented herewith may stimulate other similar studies not only in academia but also in the industry, in an effort to provide a better understanding of operational factors responsible for significant variations in successive calibration data for the same storage device.
• The tables, frequency distribution, graphs and figures presented, provides vital insights on data trends and variation in tank calibration data for successive calibration exercise.
• Access to this data will provide a platform, and basis for extensive investigation towards developing elaborate data models; both qualitative and quantitative, that will enable the development of an improved stock management system.
• This dataset may serve as an opportunity for collaborative research on related works, both locally and across the globe.

## 1 Data

In fuel depot operations, fuels such as diesel, petrol, jet fuel and so forth are transported and distributed by fuel tankers from shore depots to various fuel stations, and dispensing depots in Nigeria [1], so that consumers can have easy access to purchase needed fuel (Premium motor spirit, Kerosene and Automotive Gas Oil). To ensure accountability, as the fuel is transported, various stock keeping and inventory models are usually deployed along the value chain [2]. Typically, the actual volumetric capacity of the mobile tanks are determined using a manual or liquid calibration method to create a standard table to relate the height or depth measured to some volume specific to the tank [3]. Subsequently, a non-reactive paste is applied on a dipping stick or tape, and the stick is lowered into the storage tank to determine the fuel height from the tank base. Using the standard chart already created, the height is converted to fuel volume, and this is documented in the appropriate stock report. The report is periodically submitted to the stock department of the company for processing. A major challenge with stock keeping is the accuracy of the calibration charts. The calibration method; whether wet or dry influences the calibration accuracy [4], [5], [6]. Also, the calibration procedure and the experience of the calibration team also affect the accuracy of the calibration chart data. The training, skill and concentration of the dip stick user during fuel height measurement can affect the observed reading [7], [8]. In some countries, automated, mobile fuel truck, tank content volume measurement is available but this is not the norm in Nigeria. Stock reconciliation is therefore a major challenge in downstream petroleum companies. Labour relation issues such as Incidences of staff salaries being deducted or a staff being fired on allegations of fuel theft due to stock imbalance is not uncommon. Although, in some cases these allegations might be valid but more often than not, inaccuracies may be attributed to calibration issues. The data contained in the attached supplementary spreadsheet file, shows the calibration chart parameters of a cylindrical mobile fuel tank using a manual calibration method; for two different calibration exercises performed within a three year period on the same tank. Table 1, Table 2, Table 3, Table 4, Table 5, Table 6, Table 7, Table 8, Table 9 show comparatively the statistical analysis for the two dataset, that is Calibration Dataset A and Calibration Dataset B.

**Table 1:** Descriptive statistics of calibration chart parameters.

	**Cumulative volume – A**	**Cumulative volume – B**	**Incremental volume – A**	**Incremental volume – B**	**Deviation**
**Count**	1412	1412	1412	1412	1412
**Mean**	8809.6936	8635.5909	13.0071	12.8606	174.1027
**Min**	0	0	8.833	7.304	0
**Max**	18,352.963	18,146.267	24.606	14.612	303.94
**Range**	18,352.963	18,146.267	15.773	7.308	303.94
**Variance**	30,694,394.45	30,140,184.74	3.7251	3.5135	3667.3092
**Standard Deviation**	5540.2522	5490.0077	1.9301	1.8744	60.5583
**Standard Error of Mean**	147.4389	146.1018	0.0514	0.0499	1.6116
**Median**	8733.9175	8572.115	13.88	13.631	159.49
**Mode**	0.0000*	0.0000*	14.277	13.234	155.8140*

* Multiple modes exist. The smallest value is shown.

**Table 2:** Tests of model effects.

	**Type III**		
**Source**	**Wald chi-square**	**d*f***	**Sig.**
**A**			
**(Intercept)**	5.987	1	0.014
**Cum. Vol_A (L)**	1,519,092.497	1	0
**Increment_A L/mm**	1006.637	1	0
**B**			
**(Intercept)**	319.115	1	0
**Cum. Vol_B (L)**	2,685,306.725	1	0
**Increment_B L/mm**	3921.159	1	0

Dependent Variable: Dip (mm).

Model: (Intercept), Cum. Vol (L), Increment L/mm.

**Table 3:** Case processing summary.

	***N***	**Percent**
**Included**	1412	100.00%
**Excluded**	0	0.00%
**Total**	1412	100.00%

**Table 4:** Omnibus test.

**Likelihood ratio chi-square**	**d*f***	**Sig.**
**A**		
10,204.642	2	0
**B**		
11,223.776	2	0

Dependent Variable: Dip (mm).

Model: (Intercept), Cum. Vol (L), Increment L/mm^a^.

a Compares the fitted model against the intercept-only model.

**Table 5:** Parameter estimates.

				**95% Wald confidence interval**					**Hypothesis test**				
**Parameter**	***B***	**Std. error**		**Lower**		**Upper**			**Wald chi-square**		**d*f***		**Sig.**
**A**													
**(Intercept)**	-5.006	2.046		-9.016		-0.996			5.987		1		0.014
**Cum. Vol_A (L)**	0.073	5.90E-05		0.073		0.073			1,519,092		1		0
**Increment_A L/mm**	5.373	0.1694		5.041		5.705			1006.637		1		0
**(Scale)**	120.727^a^	4.5436		112.142		129.969							
**B**													
**(Intercept)**	-26.977	1.5102		-29.937		-24.018			319.115		1		0
**Cum. Vol_B (L)**	0.073	4.44E-05		0.073		0.073			2,685,307		1		0
**Increment_B L/mm**	8.137	0.1299		7.882		8.392			3921.159		1		0
**(Scale)**	58.661^a^	2.2077		54.489		63.151							

Dependent Variable: Dip (mm).

Model: (Intercept), Cum. Vol (L), Increment L/mm.

a Maximum likelihood estimate.

**Table 6:** Goodness of fit for the generalized linear model.

	Value	d*f*	Value/d*f*
**A**			
**Deviance**	170,466.929	1409	120.984
**Scaled Deviance**	1412	1409	
**Pearson Chi-Square**	170,466.929	1409	120.984
**Scaled Pearson Chi-Square**	1412	1409	
**Log Likelihood**^b^	-5387.776		
**Akaike׳s Information Criterion (AIC)**	10,783.553		
**Finite Sample Corrected AIC (AICC)**	10,783.581		
**Bayesian Information Criterion (BIC)**	10,804.564		
**Consistent AIC (CAIC)**	10,808.564		
**B**			
**Deviance**	82,828.747	1409	58.785
**Scaled Deviance**	1412	1409	
**Pearson Chi-Square**	82,828.747	1409	58.785
**Scaled Pearson Chi-Square**	1412	1409	
**Log Likelihood**^b^	-4878.209		
**Akaike׳s Information Criterion (AIC)**	9764.419		
**Finite Sample Corrected AIC (AICC)**	9764.447		
**Bayesian Information Criterion (BIC)**	9785.43		
**Consistent AIC (CAIC)**	9789.43		

Dependent Variable: Dip (mm).

Model: (Intercept), Cum. Vol (L), Increment L/mm^a^.

a Information criteria are in smaller-is-better form.

b The full log likelihood function is displayed and used in computing information criteria.

**Table 7:** Linear regression model summary.

**Model**	***R***	***R* square**	**Adjusted *R* square**	**Std. error of the estimate**
**A**				
1	1.000^a^	0.999	0.999	10.999288
**B**				
1	1.000^a^	1	1	7.667169

a Predictors: (Constant), Increment L/mm, Cum. Vol (L).

**Table 8:** ANOVA.

**Model**		**Sum of squares**	**d*f***	**Mean square**	***F***	**Sig.**
**A**						
1	Regression	234,426,626	2	117,213,313	968,830.5	0.000^a^
	Residual	170,466.929	1409	120.984		
	Total	234,597,093	1411			
**B**						
1	Regression	234,514,264	2	117,257,132.1	1,994,661	0.000^a^
	Residual	82,828.747	1409	58.785		
	Total	234,597,093	1411			

a Predictors: (Constant), Increment L/mm, Cum. Vol (L).

**Table 9:** Coefficients.

		**Unstandardized coefficients**		**Standardized coefficients**		
**Model**		***B***	**Std. error**	**Beta**	***t***	**Sig.**
**A**						
1	(Constant)	-5.006	2.048		-2.444	0.015
	Cum. Vol_A (L)	0.073	0	0.988	1231.205	0
	Increment_A L/mm	5.373	0.17	0.025	31.694	0
**B**						
1	(Constant)	-26.977	1.512		-17.845	0
	Cum. Vol_B (L)	0.073	0	0.979	1636.949	0
	Increment_B L/mm	8.137	0.13	0.037	62.553	0

## 2 Experimental design, materials and methods

**Fig. 1:**
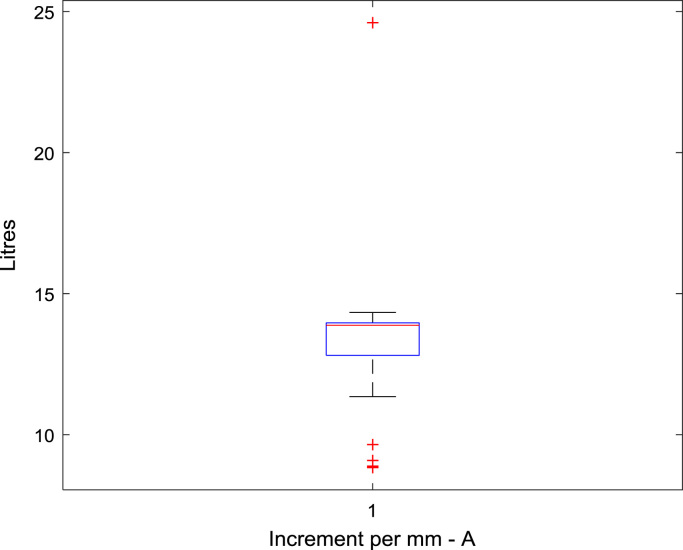
A box plot of incremental volume for calibration A.

**Fig. 2:**
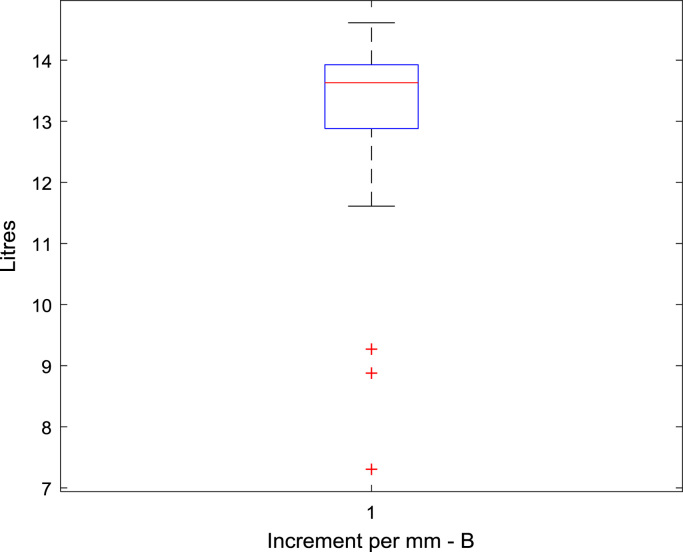
A box plot of incremental volume for calibration B.

**Fig. 3:**
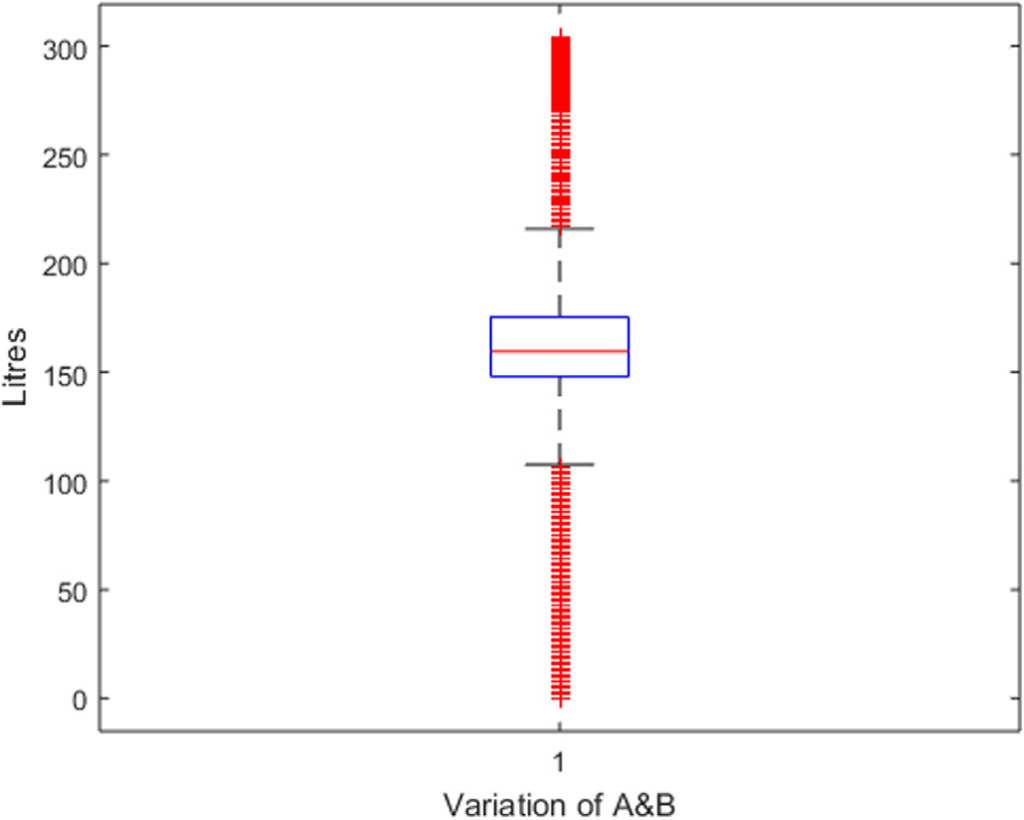
A box plot of cumulative volume variation between calibration A and B.

**Fig. 4:**
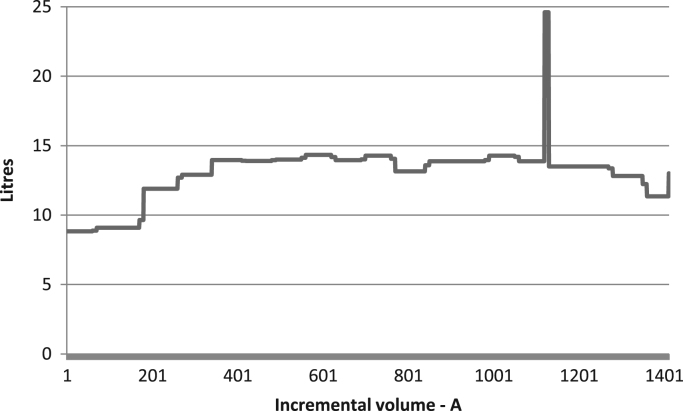
Incremental volume with increasing tank dip for calibration A.

Raw data was extracted from two calibration charts for a mobile fuel storage tank. Three key parameters were extracted from each chart, and these are: Dip (mm), Cumulative Volume-A (L), the Incremental Volume-A (L) for calibration chart A, and Dip (mm), Cumulative Volume-B (L), the Incremental Volume-B (L) for calibration chart B. Another parameter termed Deviation (L) is created using the difference between the Cumulative Volume-A and the Cumulative Volume-B. The two datasets were analysed to identify variations in data pattern, and most importantly, to reflect any difference in the relationship between the Dip as the target parameter, and the Cumulative and Incremental Volume as the predictors, for the two calibration data sets. Fig. 1, Fig. 2, Fig. 3 show the boxplots of three parameters: the volume increment per mm for Dataset A, volume increment per mm for Dataset B, and the difference between the cumulative volume for Dataset A and B. Fig. 4, Fig. 6 show the line plots for all the data points of the three parameters.

**Fig. 5:**
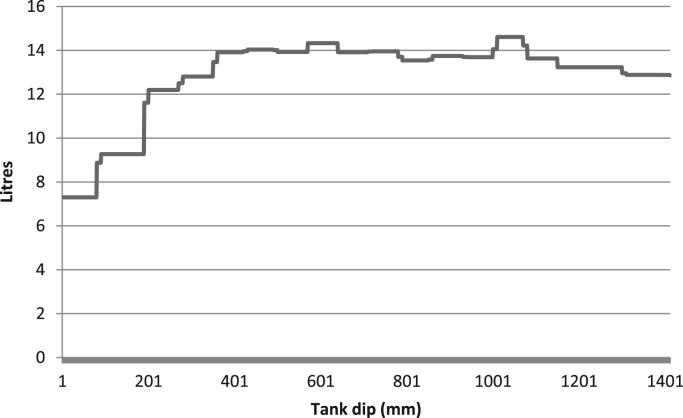
Incremental volume with increasing tank dip for calibration B.

**Fig. 6:**
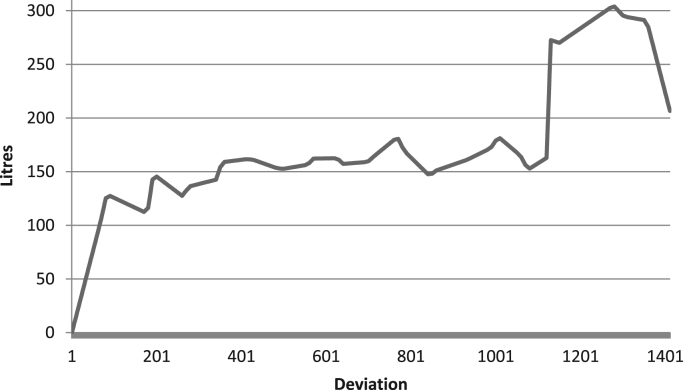
Volumetric variation of calibration A and B with increasing tank dip.
